# Design, Optimization, and Modeling Study of Ultrasound-Assisted Extraction of Bioactive Compounds from Purple-Fleshed Sweet Potatoes

**DOI:** 10.3390/foods13101497

**Published:** 2024-05-12

**Authors:** Bárbara Avancini Teixeira, Eliana Alviarez Gutiérrez, Mariane Sampaio da Silveira de Souza, Thaís Caroline Buttow Rigolon, Evandro Martins, Fernando Luiz Pellegrini Pessoa, Márcia Cristina Teixeira Ribeiro Vidigal, Paulo Cesar Stringheta

**Affiliations:** 1 Departamento de Tecnologia de Alimentos, Universidade Federal de Viçosa, Viçosa 36570-900, MG, Brazil; mariane_sss23@hotmail.com (M.S.d.S.d.S.); tcbuttow@yahoo.com.br (T.C.B.R.); evandromartins@ufv.br (E.M.); marcia.vidigal@ufv.br (M.C.T.R.V.); pstringheta@gmail.com (P.C.S.); 2 Campus Piatã, Centro Universitário SENAI CIMATEC, Salvador 41650-010, BA, Brazil; fernando.pessoa@fieb.org.br; 3 Instituto de Investigación para el Desarrollo Sustentable de Ceja de Selva, Universidad Nacional Toribio Rodríguez de Mendoza de Amazonas, Chachapoyas 01001, AM, Peru; eli2099@hotmail.com

**Keywords:** ultrasonic extraction, optimization, kinetic modeling, anthocyanins, phenolic compounds

## Abstract

This study focuses on optimizing the ultrasound-assisted extraction (UAE) of bioactive compounds from purple-fleshed sweet potatoes (PFSP) for potential use as natural colorants. Factors such as time, temperature, and solid-to-liquid ratio were varied using a Box–Behnken Design. The optimal conditions were determined as 75 min, 70 °C, and a 1:15 m/v solid-to-liquid ratio, resulting in 18.372 mg/100 g total anthocyanin (TA) and 151.160 mg GAE/100 g total phenolic content (TPC). The validation yielded 18.822 mg/100 g for total anthocyanin and 162.174 mg GAE/100 g for total phenolic content, showing a 7% difference from predictions. UAE significantly increased TA extraction by 81% and TPC by 93% compared with the conventional method, with a notable reduction in process time from 24 h to 75 min. Additionally, three kinetic models were tested to compare extraction mechanisms, confirming the efficiency of UAE for PFSP bioactive compound recovery. This study proposes the UAE technique as a highly effective means of extracting bioactive compounds from PFSP, offering promising applications across multiple industries.

## 1. Introduction

The sweet potato (*Ipomoea batatas* L.), belonging to the *Convolvulaceae* family, stands out as one of the most significant root crops globally, renowned for its simplicity in cultivation and management. In recent times, it has garnered considerable attention in research circles, owing to its distinctive nutritional and functional attributes. This tuberous root boasts a wealth of carbohydrates such as starch, alongside crude protein, dietary fiber, vitamins, minerals, and bioactive compounds like polyphenols and pigments. Sweet potatoes are distinguished by the color of their peel and flesh, with variants ranging from white to yellow, orange, and purple. Notably, the purple-fleshed sweet potato (PFSP), a specialized cultivar, is particularly abundant in anthocyanins [1,2].

Anthocyanins, a group of naturally occurring polyphenolic compounds, are pigments found abundantly in fruits, legumes, grains, and vegetables. They can serve as natural colorants in pharmaceuticals, cosmetics, and food and beverages industries while also acting as powerful antioxidants against oxidative stress. This property contributes to their role in protecting against various chronic ailments like cardiovascular diseases, degenerative disorders, and cancer [3,4,5,6]. Despite the prevalence of synthetic colorants in various industries, they pose health risks such as allergies and intolerances, particularly in children. Consequently, there is a gradual phasing out of synthetic colorants from the market, with natural alternatives, like anthocyanins, gaining traction as viable replacements [7,8,9]. 

As a negative point, anthocyanins can be easily degraded into colorless or brown-colored compounds during processing and storage, which limits their use as a commercial colorant. Usually, the stability of anthocyanins depends on temperature, light, enzymes, metal ions, sugars, ascorbic acid, oxygen, and the presence of other phenolic compounds. Nowadays, anthocyanins present in PFSP are recognized as more stable than the pigments of strawberries, blueberries, cranberries, red cabbage, perilla, and other plants, due to the anthocyanins showing high pH stability, thermostability, and antioxidant activity. Therefore, PFSP has been recognized as a promising alternative for providing stable anthocyanins, serving as a natural colorant in industrial applications [10,11].

The production of natural colorants begins with extracting them from vegetable matrices, but large-scale production necessitates understanding how extraction conditions affect the bioactive ingredient’s properties [12]. Conventional methods such as maceration are hindered by lengthy extraction periods and significant solvent usage, prompting the emergence of assisted extraction techniques. In the last decade, ultrasound-assisted extraction (UAE) has emerged as a key technology for sustainable and green extraction, offering high efficiency in terms of performance, reduced extraction times, and decreased solvent usage [13]. In this sense, UAE provides an economically viable alternative by overcoming conventional extraction limitations. The ultrasonic enhancement of extraction is attributed to various factors like cell destruction and improved solvent penetration [14]. Despite its potential, optimizing UAE parameters is crucial as different plant matrices require specific conditions for better extraction yields [15,16,17]. Moreover, alongside the advancement of the extraction technique, there must be a concurrent focus on enhancing mathematical models, comprehending their kinetics and mechanisms for improved efficacy, which enables the generalization of the experimental results for further scale-up approaches. Some of these models are based on physical concepts as in film theory, unsteady-state diffusion through plant material, and Ponomaryov’s equation [18].

In this study, the objective was to design, optimize, and model the extraction process of bioactive compounds from PFSP, focusing on anthocyanins and phenolics. The investigation aimed to analyze the impact of three variables in ultrasound-assisted extraction (UAE): time (minutes), temperature (°C), and solid-to-liquid ratio (mass/volume). Additionally, the study compared the efficiency of this method with conventional extraction techniques. Due to its superior stability compared with other anthocyanin sources, PFSP enables the efficient extraction of this pigment, facilitating the production of a commercially viable natural colorant as a substitute for synthetic ones. Additionally, a morphological examination using scanning electron microscopy was conducted, along with the application of three distinct physical kinetic models for both conventional and UAE extraction methods.

## 2. Materials and Methods

### 2.1. Chemicals and Reagents

Folin–Ciocalteu phenol reagents were purchased from Sigma-Aldrich Co. (St. Louis, MO, USA), while the gallic acid phenolic standard was obtained from Carlo Erba (Milano, Italy). All chemicals and reagents, unless specified, were of analytical reagent grade, and ultrapure water was used in the preparation of all solutions.

### 2.2. Plant Material 

Purple-fleshed sweet potatoes grown in Ribeirão Corrente, SP, Brazil (latitude: 20°27′25″ south, longitude 47°35′25″ west), were utilized. Tuberous roots were harvested, meticulously chosen, sanitized, frozen, packaged, and subsequently stored in a standard freezer at −20 °C until analysis. The moisture content, determined by drying to constant mass at 105 °C, was approximately 72%.

### 2.3. Design of Experiments 

The Box–Behnken Design (BBD) was chosen to design and optimize the conditions of UAE for recovery of TA and TPC from PFSP [17,19]. Prior to this, preliminary investigations (data not presented) were conducted to establish the optimal high and low values for the levels of the three independent variables: time, temperature, and solid-to-liquid ratio. These variables were evaluated at three levels: −1, 0, and +1, representing low, medium, and high values, respectively. This resulted in 17 combinations, including five repetitions of the central point, as outlined in Table 1. The experiments were performed randomly. Total anthocyanin (TA) and total phenolic compound (TPC) yields served as response variables. The acquired experimental data underwent a regression analysis (*p* < 0.05), wherein they were subjected to fitting procedures utilizing a quadratic polynomial model represented by Equation (1). The regression coefficients pertinent to this analysis were derived via multiple linear regression techniques:

(1) $$Y_{i}=\beta_{0}+\sum \beta_{i}X_{i}+\sum \beta_{ii}X_{i}^{2}+\sum \beta_{ij}X_{i}X_{j}$$

where *Y_i_* represents the dependent variable and *X_i_* denotes the independent variable. *Β*_0_, *β_i_*, *β_ii_*, and *β_ij_* represent the regression coefficients for the intercept (constant), linear effect, quadratic effect, and interaction effects, respectively. *X_i_* and *X_j_* refers to the levels of the independent variables under examination, where *i* ≠ *j* [4,20,21].

Desirability profiles were utilized to concurrently pinpoint the maximum values of the independent variables under investigation, with the goal of attaining the highest content of total anthocyanin (TA) and total phenolic content (TPC). This method facilitated the determination of optimal conditions for ultrasound-assisted extraction (UAE). Utilizing this optimal combination, predicted values were generated for the extraction of TA and TPC, which were subsequently experimentally validated to verify the efficacy of the approach.

Desirability profiles offer several advantages in process optimization. They facilitate multi-objective optimization by concurrently optimizing multiple responses or objectives, resulting in a well-balanced solution. Additionally, their flexibility and adaptability render them suitable for addressing various optimization challenges across industries. Furthermore, they provide a visual representation of trade-offs between factors, aiding decision makers in comprehending results. Moreover, desirability profiles promote efficient resource utilization by pinpointing optimal operating conditions, thereby reducing waste and enhancing sustainability. They also enable robustness assessment, ensuring consistent performance under different conditions. Overall, the desirability profile approach enhances efficiency, effectiveness, and reliability in process optimization, making it a valuable tool for improving product quality and operational performance [19,20].

### 2.4. Ultrasound-Assisted Extraction 

The purple-fleshed sweet potatoes underwent grinding using a common mixer (PMX600, Philco, Manaus, AM, Brazil). Subsequently, a mixture consisting of 5 g of ground plant material and a volume of aqueous solvent of ethanol:water at a ratio of 70:30 (*v*/*v*) was prepared. The volume of the solvent ranged between 25, 50, and 75 mL, depending on the solid-to-liquid ratio determined by the experimental design (see Table 1). In order to improve the stability of anthocyanins within the plant material during extraction, the system was acidified to a pH of 2.00 ± 0.10 using concentrated HCl [22].

An ultrasonic bath with a power rating of 800 W (Elmasonic TI-H-10, Elma, Singen, Germany), featuring a tank capacity of 8.6 L, was employed. The crude phenolic extracts underwent processing at a consistent frequency of 25 kHz, with the ultrasonic power amplitude being set at 50%. The equipment was equipped with a temperature control. UAE was conducted under controlled conditions using the combination of variables generated by the experimental design. The plant matrix:solvent systems were subjected to the respective variables: X1—extraction time, ranging from 5 to 75 min; X2—temperature, ranging from 30 to 70 °C; and X3—solid-to-liquid ratio, ranging from 1:5 to 1:15 g/mL (as detailed in Table 1). After ultrasound-assisted extraction, the crude extracts underwent vacuum filtration through Whatman filter paper no. 1, and their volume was adjusted to a standardized level using 70% ethanol (*v*/*v*). Subsequently, these extracts were stored in volumetric flasks at −20 °C until further analysis [23].

### 2.5. Conventional Extraction

The conventional extraction process followed the methodology proposed by Rocha et al. [22] using a ratio of 1:10 (m/v) of ground vegetable material to solvent (70% ethanol, *v*/*v*). This mixture was acidified to pH 2.00 ± 0.10 with concentrated HCl and refrigerated for 24 h at 5 ± 1 °C. Subsequently, the crude extracts were also vacuum-filtered through Whatman filter paper no. 1 and stored in amber vials in a conventional freezer at −20 °C until further analysis. The conventional extraction procedure was conducted in triplicate.

### 2.6. Kinetics and Modeling of Extraction 

To assess potential enhancements in the efficiency of extracting target compounds, a comparative kinetics investigation was conducted among the optimized ultrasound-assisted extraction method and the conventional process, drawing upon research by Veličković et al. [24] and Dias et al. [25]. Samples were subjected to the optimized UAE conditions (temperature and solid-to-liquid ratio) for durations of 0, 25, 50, 75, 100, 150, 200, and 250 min. Likewise, a study was conducted under conventional extraction conditions to compare the kinetics of both processes. After extraction, crude phenolic extracts were likewise filtered through Whatman filter paper no. 1, and their volume was standardized using 70% ethanol (*v*/*v*). Subsequently, the extracts were stored at −20 °C until further analysis. 

The recovery of the target compounds extracted in the kinetic curve were employed to model the process, utilizing three physical theories: film theory, theory of unsteady diffusion through plant material, and Ponomaryov’s empirical equation [18,26]. The equations and their linearized forms are presented in Table 2.

The recovery of the target compound in the saturated liquid extract at equilibrium (*c_seq_*) was derived from the research of Veličković et al. [24] with some adjustments. Initially, 5 g of plant material in 75 mL of 70% *v/v* ethanol underwent an extraction cycle in the ultrasonic bath under the optimized conditions of UAE. The liquid portion of the extract was obtained by vacuum filtration, and a new portion of plant material (5 g) was added for a new extraction cycle to be run. A total of three extraction/filtration cycles were conducted for the content of the target compound to reach equilibrium. The *c_seq_* values obtained for TA and TPC were 8.923 ± 0.37 and 252.00 ± 0.69 mg/L, respectively.

The initial determination of TA and TPC content in plant material (*q*_0_) was established following the methodology outlined by Veličković et al. [24] with slight adjustments. In this case, first, 5 g of plant material in 75 mL of 70% *v/v* ethanol underwent an extraction cycle in the ultrasonic bath under the optimized conditions of UAE. The liquid extract was separated through vacuum filtration, followed by the addition of 50 mL of fresh solvent (equivalent to 2/3 of the previous volume) to the residual plant material, allowing for a subsequent extraction cycle. This process was repeated for a total of three extraction/filtration cycles until reaching equilibrium. The resulting q0 values for TA and TPC were determined to be 54.333 ± 3.43 and 305.521 ± 4.39 mg/100 g, respectively.

### 2.7. Total Anthocyanin (TA) Analysis

The content of total anthocyanin of purple-fleshed sweet potato extracts was determined following the method outlined by Fuleki and Francis [27] with slight modifications. A sample aliquot was diluted in a volumetric flask using a solvent consisting of 85:15 *v/v* ethanol:HCl. The absorbance was then measured at 535 nm using a UV-Vis spectrophotometer (Bel Engineering, Model UV-51, Monza, Italy). The determination of total anthocyanin (TA) in the extract was determined directly by Equation (2) and expressed as mg per 100 g of sample (mg/100 g).

(2) $$C=\frac{Abs\times MM}{\varepsilon \times b}$$

where *C* represents the recovery of anthocyanins in g/L; *Abs* denotes the absorbance read at 535 nm; *MM* signifies the molar mass in g/mol, where cyanidin-3-glycoside MM 449.2 g/mol was used; *ε* indicates the molar absorptivity in a specific solvent in L/mol.cm (in this case, 26900); and b represents the cuvette thickness, 1 cm.

### 2.8. Total Phenolic Content (TPC) Analysis

The total phenolics content was determined following the method developed by Singleton and Rossi [28]. A volume of 0.6 mL of the diluted extract was combined with 3.0 mL of 10% (*v*/*v*) Folin–Ciocalteu reagent and 2.4 mL of 7.5% sodium carbonate (m/v) in a volumetric flask. This mixture was shielded from light and allowed to stand for 1 h at room temperature. Subsequently, its absorbance was measured at 760 nm using a UV-Vis spectrophotometer (Bel Engineering, Model UV-51, Monza, Italy). The quantification of total phenolic content (TPC) was determined using the standard curve of gallic acid (with an R^2^ value of 0.991), and the results were expressed in milligrams of gallic acid equivalent per 100 g of sample (mg GAE/100 g).

### 2.9. Total Starch Content

The total starch content in the raw material was determined according to method 76-13 (AACC, 2009) using the total starch assay kit (Megazyme International, Wicklow, Ireland). The total starch content found in PFSP was 10.872 ± 0.15 g/100 g.

### 2.10. Validation of Optimized UAE and Comparative Study of Extraction Efficiency

The optimal combination of independent variables (time, temperature, and solid-to-liquid ratio) for achieving maximum extraction of TA and TPC from purple-fleshed sweet potato was determined using desirability profiles. Subsequently, the contents of TA and TPC were experimentally obtained to validate the method based on this optimal combination. Furthermore, a comparative study was conducted between the optimized conditions of ultrasound-assisted extraction and conventional extraction to assess the efficiency of ultrasound in enhancing the recovery of TA and TPC from PFSP.

### 2.11. Scanning Electron Microscopy (SEM)

To enhance the understanding of extraction process variances, the microstructures of untreated purple-fleshed sweet potato, as well as those subjected to conventional and ultrasound-assisted extraction (UAE), were examined using a scanning electron microscope (JSM-6010 LA, Jeol, Tokyo, Japan). Initially, samples were affixed to metal supports (stubs) using carbon tape and subsequently coated with a thin layer of gold utilizing a sputtering apparatus (Quorum Q150R, Quorum Technologies Ltd., East Sussex, UK). The samples were then scanned using SEM under partial vacuum conditions at 10 kV.

### 2.12. Statistical Analysis

The statistical software Statistica 7.0 (StatSoft Inc., Tulsa, OK, USA) was employed to analyze the data obtained from the Box–Behnken design (BBD) by regression analysis (*p* < 0.05). It was used to assess the significance of regression coefficients (*p* < 0.05) as well as optimize the ultrasound-assisted extraction process conditions based on desirability profiles. 

The BBD data were analyzed using independent variables in a parameterized manner, which means that these variables were adjusted so that their effects could be compared more accurately. However, the data were not analyzed using power transformation. Instead, a direct approach was chosen to facilitate data analysis.

All results are presented as the mean ± standard deviation. Significant differences among different conditions were determined using analysis of variance (ANOVA), with a significance level set at *p* < 0.05.

## 3. Results and Discussion

### 3.1. Design and Optimization of Ultrasound-Assisted Extraction

Crude phenolic extracts from purple-fleshed sweet potato were obtained through ultrasonic treatment, investigating the following variables: extraction time (*X*_1_) ranging from 5 to 75 min, ultrasonic bath temperature (*X*_2_) ranging from 30 to 70 °C, and solid-to-liquid ratio (*X*_3_) ranging from 1:5 to 1:15 g/mL. This comprised a total of 17 experimental runs. Under these circumstances, the recovery of total anthocyanin (TA) ranged from 6.750 to 16.424 mg/100 g, while the recovery of total phenolic content (TPC) ranged from 53.366 to 130.661 mg GAE/100 g (Table 1). BBD was employed to assess the linear, quadratic, and interaction effects of the variables under investigation on the respective response variables. Table 3 presents the regression analysis coefficients and *p*-values for the responses related to TA and TPC.

During the regression analysis for the TA response variable (Table 3), it was observed that the linear time (*X*_1_) was the most significant effect on the model (*p* < 0.01), followed by the effect of the interaction of linear time with the linear volume of solid-to-liquid ratio (*X*_1×3_), the negative effect of the quadratic volume of solid:liquid ratio (*X*_3_^2^), and the effect of linear temperature (*X*_2_), both with *p* < 0.05. In addition, for the TPC response variable (Table 3), the effect of linear time (*X*_1_) and the effect of the linear volume of the solid:liquid ratio (*X*_3_) were the most significant for the model (*p* < 0.01)—followed by the negative effect of quadratic temperature (*X*_2_^2^), the effect of the interaction of linear time with the linear volume of solid: liquid ratio (*X*_1_*X*_3_), the effect of quadratic time (*X*_1_^2^), and the effect of linear temperature (*X*_2_)—both with *p* < 0.05.

The regression analysis revealed that the studied factors were fitted to the second-order polynomial regression model (Equation (1)), indicating significant F test values, a non-significant lack of fit, and R^2^ values equal to 0.978 for TA and 0.981 for TPC. The high values of the coefficient of determination (R^2^ > 0.90) show that the regression equations used for TA (Equation (3)) and TPC (Equation (4)) fitted the experimental data. It is important to highlight that while the generated polynomials (Equations (3) and (4)) may not fully capture the underlying phenomena of the ultrasonic extraction process, they are still valuable for assessing the effects of time, temperature, and solid-to-liquid ratio on the recovery capacity of anthocyanins and phenolic compounds from PFSP, as well as for predicting these contents. The Supplementary Materials comprises ANOVA tables, as well as response surface and contour plots for all variables investigated in the study (Table S1 and Figures S1 and S2).

(3) $$Y_{TA}=11.079+5.613X_{1}+1.961X_{2}-1.455X_{3}^{2}+4.129X_{1}X_{3}$$

(4) $$Y_{TPC}=96.425+34.323X_{1}+9.608X_{2}+39.118X_{3}+6.727X_{1}^{2}-13.891X_{2}^{2}+15.239{X_{1}X}_{3}$$

**Figure 1:**
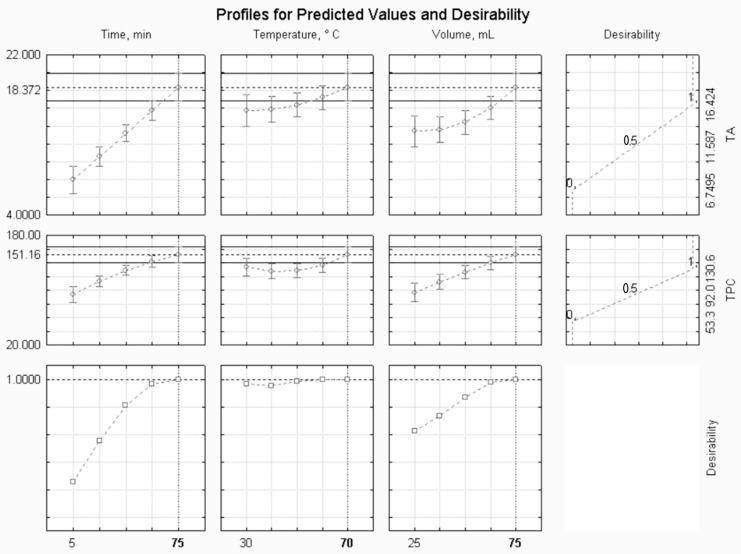
Desirability profile for TA (mg/100 g) and TPC (mg GAE/100 g).

Desirability profiles, on a scale from 0 to 1, were utilized to simultaneously optimize the three variables under study (time, temperature, and solid-to-liquid ratio) to achieve the maximum contents of TA and TPC. As depicted in Figure 1, the optimal combination for both TA and TPC was observed at a time of 75 min, a temperature of 70 °C, and a solid-to-liquid ratio of 1:15 (m/v), equivalent to 75 mL of 70% ethanol solvent (*v*/*v*) for 5 g of mashed PFSP. This combination is expected to yield a TA content of 18.372 mg/100 g and a TPC content of 151.160 mg GAE/100 g. Upon validation of these optimized conditions, experimental values of 18.822 ± 1.59 mg/100 g for TA and 162.174 ± 12.44 mg GAE/100 g for TPC were observed. These values are only around 3% higher for TA and 7% higher for TPC compared with those predicted by the desirability profile.

This study indicates that the extraction of TA and TPC increased with the rise in all three studied factors. Notably, a temperature of 70 °C was found to be the most favorable condition for achieving the highest efficiency in both TA and TPC extraction (Figure 1). This observation aligns with the general principle in solid–liquid extraction processes, where higher temperatures typically lead to a greater recovery of bioactive compounds [15]. This phenomenon can be attributed to the increase in solubility and diffusion coefficients of the compounds extracted from the plant matrix as temperature rises. Additionally, the decrease in solvent viscosity at higher temperatures facilitates mass transfer within the system [29].

The solid-to-liquid ratio (m/v) emerged as a crucial factor in enhancing the recovery of both TA and TPC. In both instances, the highest yield was achieved at the highest level of solid-to-liquid ratio assessed, which was 1:15 m/v (Figure 1). Increasing the volume of solvent can enhance efficiency by facilitating solute access to the solvent. This is because the solvent acts as the liquid medium where ultrasonic acoustic cavitation occurs, a process that generates various mechanical effects, including particle collision and cell disintegration. Consequently, larger solvent volumes can promote the occurrence of these phenomena [4]. In their study, Liao et al. [30] examined the extraction of monomeric anthocyanins and phenolics from purple eggplant peels. They investigated varying the solid-to-liquid ratio (using 50% aqueous ethanol) from 1:10 to 1:60 m/v. Their findings indicated that increasing the ratio above 1:30 m/v improved phenolics recovery, whereas the variation in ratio had less impact on anthocyanin recovery. Consequently, the authors suggest that considerations regarding the necessity for a high dilution factor should weigh the economic factors of energy and operational costs alongside sustainability requirements when making decisions. 

Furthermore, the extraction process was optimized by varying the duration between 5 and 75 min. According to the desirability profiles (Figure 1), it was noted that the impact of time was markedly significant for the recovery of both TA and TPC, with the highest extraction efficiency being observed at 75 min. Over the past decade, ultrasound-assisted extraction has been extensively documented to enhance the extraction rates of target compounds while reducing extraction time [4,15,22,31,32]. In this sense, in a recent study developed by Chua et al. [33], the use of ultrasound as pre-treatment for just 30 min also enhanced the extraction of anthocyanins and phenolics from jaboticaba peels by 61% and 75%, respectively.

Usually, anthocyanins are highly sensitive to factors such as temperature, pH, oxygen, and water activity, which often limits their yield in the extraction process [4]. Moreover, when compared with conventional extraction, the cavitation and mechanical effects produced by ultrasound may destroy the structure of anthocyanins in UAE [34]. Some studies have already investigated the greater stability of PFSP anthocyanins when compared with other sources such as strawberries, blueberries, cranberries, red cabbage, perilla, and others and have attributed it to several factors like the presence of highly acylated anthocyanins such as cyanidin 3-sophoroside-5-glucoside and peonidin 3-sophoroside-5-glucoside [35], others polyphenols such as cinnamoyl quinic acids [36], protein-bound anthocyanin compounds [37], and co-pigmentation [9].

**Figure 2:**
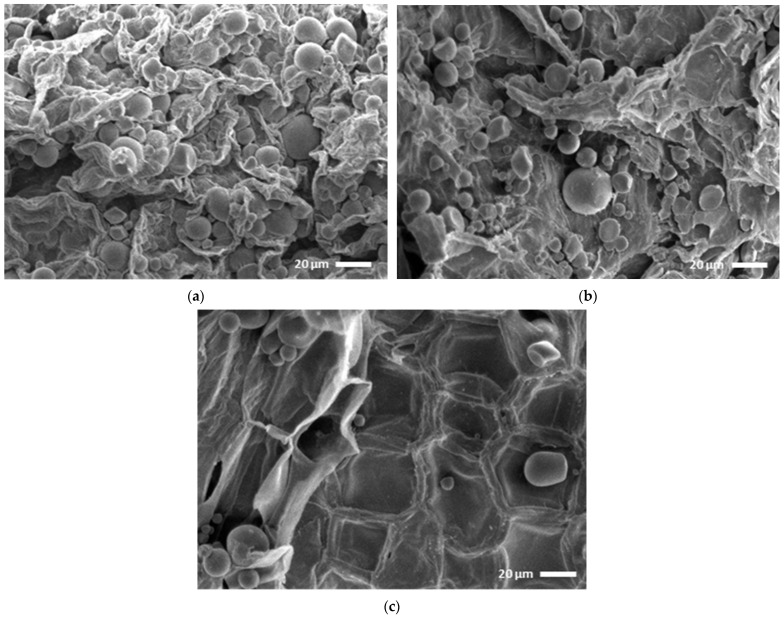
Scanning electron micrograph of (**a**) untreated purple-fleshed sweet potato; (**b**) conventional extraction; (**c**) UAE.

Another hypothesis for the greater stability of anthocyanins present in PFSP when compared with other sources of anthocyanins may be due to the starch content (10.872 ± 0.15 g/100 g) present in this raw material. The starch present in vegetables is found in granular structures, as can be seen in Figure 2, and it is formed by amylose and/or amylopectin molecules. The UAE of anthocyanins and phenolic compounds took place in a liquid and acidic medium (pH 2) at 70 °C for 75 min. Under these conditions, the starch granules underwent gelatinization (irreversible swelling) with consequent disruption of some of these structures, which can release amylose and amylopectin molecules into the liquid medium. Under acidic conditions, starch molecules can undergo depolymerization, forming, for example, dextrins, which can be complex with anthocyanins and phenolic compounds, increasing their stability [38,39,40]. However, further in-depth studies on these mechanisms are still necessary. In any case, a stable source of anthocyanins is interesting for commercial exploitation of the pigment, obtained from a natural source such as in PFSP, which can be used by the food and beverage, pharmaceutical, and cosmetics industries in a myriad of products, with the additive of having antioxidant properties, with a series of health benefits.

### 3.2. Optimized Extraction Compared with Conventional Extraction

In optimizing ultrasound-assisted extraction for both TA and TPC, the ideal simultaneous combination of variables occurs at a duration of 75 min, a temperature of 70 °C, and a solid-to-liquid ratio of 1:15 m/v. In contrast, conventional extraction operates at a temperature of 5 ± 1 °C, with a solid-to-liquid ratio of 1:10 m/v and duration of 24 h. This study demonstrates that UAE significantly enhances (*p* < 0.05) the extraction yield of TA, yielding 18.822 ± 1.59 mg/100 g compared with 10.396 ± 0.05 mg/100 g obtained via conventional extraction. Similarly, the extraction yield of TPC was notably higher with UAE, measuring 162.174 ± 12.44 mg GAE/100 g compared with 83.939 ± 6.31 mg GAE/100 g obtained through conventional extraction. UAE has thus proven to be a highly efficient technology, overcoming the limitations of conventional techniques. It increased the recovery yield of TA by approximately 81% and TPC by approximately 93%, nearly doubling the results achieved by conventional extraction. The reduction in processing time from 24 h in conventional extraction to just 75 min with UAE is particularly noteworthy. This reduction not only decreases energy consumption but also allows for greater processing capacity for vegetable raw materials [41].

The superior efficacy of UAE compared with conventional extraction stems from its utilization of ultrasonic waves, which induce cavitation. This phenomenon enhances the penetration of the solvent and accelerates the mass transfer rates of target compounds. Furthermore, ultrasonic power effectively disrupts plant cell walls, facilitating the movement of compounds into the solvent and consequently increasing the extraction yield. UAE offers several advantages, including scalability to industrial levels, low initial investments, and alignment with sustainable principles. Consequently, it emerges as a practical and sustainable alternative for extracting anthocyanins and phenolic compounds from various plant sources, including PFSP [4,32,42].

### 3.3. Comparative Study of Scanning Electron Microscopy

Presently, researchers are exploring the underlying mechanisms responsible for the positive effects of ultrasound on the extraction of bioactive compounds [23]. Chemat et al. [14] shed light on the mechanisms involved in ultrasound-assisted extraction for natural products, including fragmentation, erosion, capillarity, detexturization, and sonoporation. These mechanisms, either individually or in combination, enhance the efficiency of ultrasound by disrupting cells and facilitating mass transfer during the extraction process. To observe the effects of these mechanisms on the PFSP plant matrix, a scanning electron microscopy study was conducted. The untreated plant matrix exhibits a mostly intact surface, along with a high concentration of spherical starch granules [1] (Figure 2a). Following conventional extraction (24 h under refrigeration at pH~2), the plant matrix displays significant fragmentation, likely due to prolonged exposure to an acidic medium, and a noticeable reduction in the number of starch granules (Figure 2b). In comparison, ultrasonic extraction (75 min at 70 °C) reveals lower fragmentation, possibly attributable to the shorter extraction time. However, there is greater erosion, sonoporation, and detexturization of the plant material’s surface, along with a substantial decrease in the presence of starch granules, which may have been broken down after gelatinization (Figure 2c).

Thus, the observations indicate that the combination of ultrasonic treatment, heat, and acidic conditions led to the disruption of particle microstructures and plant cell walls, thereby facilitating the release and transportation of intracellular and/or intra-particle constituents into the extracting liquid. Consequently, the accelerated extraction rate achieved through ultrasound can be attributed to the alterations in the microstructures and morphology of PFSP particles [42,43,44]. This fact can also be observed by comparing the extraction kinetics of the processes.

### 3.4. Comparative Study of Kinetics and Modeling

**Figure 3:**
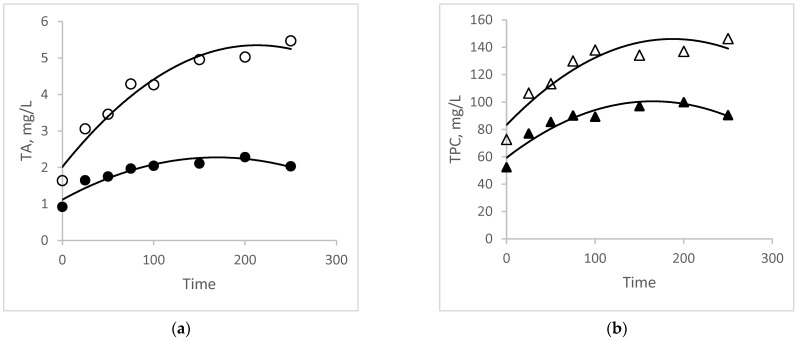
Kinetics of extraction of (**a**) TA (mg/L) and (**b**) TPC (mg GAE/L) from purple-fleshed sweet potato as a function of time (min). Legend: TA: circles; TPC: triangles; UAE: open symbols; and conventional extraction: black symbols.

When examining the extraction yield of TA and TPC over time (min) and comparing UAE with conventional extraction (Figure 3), it becomes evident that the UAE method outperforms the conventional one in terms of both shortened extraction time and increased yield. Additionally, the curve representing the UAE for the TPC response variable (Figure 3b) demonstrates higher extraction efficiency within the initial 75 min of the process, aligning with the optimal time determined in the optimization study for this compound. Beyond this timeframe, there is a certain linearity observed in the kinetic curve of TPC extraction, suggesting that phenolic extraction may have approached equilibrium at this stage of the process.

In terms of the extraction yield of TA (Figure 3a) over time (min), an uptick in TA extraction is noticeable after 75 min of extraction. This increase could be attributed to the stabilization of anthocyanins, potentially facilitated by factors such as the presence of starch in the plant matrix, among other hypotheses. Additionally, the steeper slope of the curves at the onset of the extraction process, particularly evident in the UAE curves, can be attributed to the two primary stages of this process. The initial stage involves rapid mass transfer, characterized by the penetration of the solvent into the plant cell structure and the subsequent washing of soluble constituents into the extractant solvent. Subsequently, in the second stage, there is a slower diffusion of soluble constituents through the porous structure of the residual solids into the extracting solvent, resulting in a less steep curve [26,29].

In addition to the kinetic investigation, the advancement of the UAE technique necessitates the development of mathematical models that describe the extraction process using mathematical approximations. Veličković et al. [26] proposed modeling the extraction process of bioactive substances utilizing film theory, unsteady diffusion through plant material theory, and the empirical equation of Ponomaryov (Table 2). The derivation of model equations can be found in Veljković and Milenović [45]. These models are all two-parameter models and are based on a two-stage extraction mechanism. One parameter characterizes the washing stage (b, washing coefficient), while the second parameter characterizes the slow extraction (k, slow extraction coefficient). It is important to note that these models offer more than just a fundamental understanding of a given system; they also serve as a foundation for simulation studies. Such studies allow for the extrapolation of the process to larger scales, including pilot and industrial scales [29]. In this regard, modeling and simulation studies could serve as a crucial turning point, facilitating the transition of ultrasonic technology from laboratory-scale experimentation to industrial applications.

**Figure 4:**
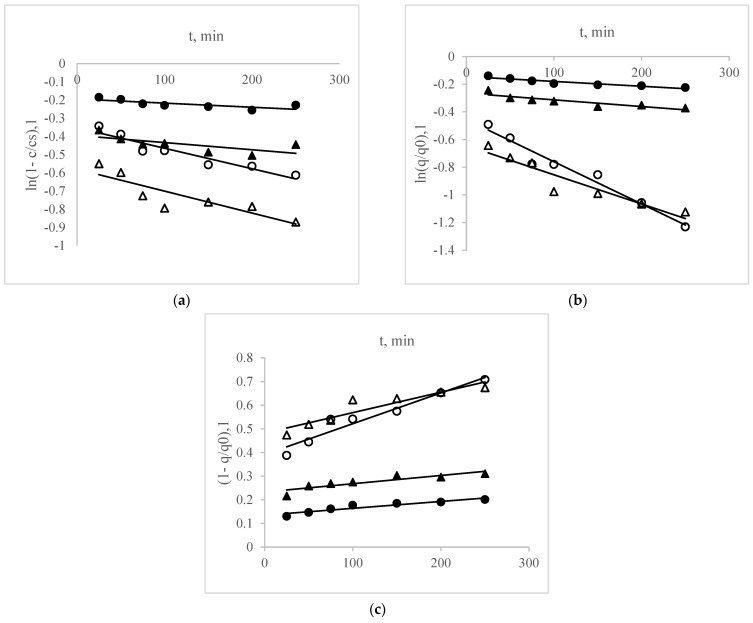
Linearized form of the kinetic equations based on (**a**) film theory, (**b**) unsteady diffusion through plant material, and (**c**) Ponomaryov’s empirical equation. Legend: TA: circles; TPC: triangles; UAE: open symbols; and conventional extraction: black symbols.

Figure 4 illustrates the linearized representations of the fundamental kinetic equations provided in Table 2. The parameters of the kinetic models were established by employing the linear regression method on the experimental data, utilizing the linearized form of the proposed kinetic equations outlined in Table 2. This process is illustrated in Table 4 and Figure 4a–c. The criteria used to evaluate how well models represent the experimental data was the coefficient of determination (R^2^). Although the tested models were originally designed to describe the conventional solid–liquid extraction, in general, the models adapted better to the ultrasonic process which, as a rule, exhibited higher R^2^ (close to or greater than 0.90) than conventional ones (smaller than 0.90). Through the higher coefficients of determination, it is also possible to observe that the models of unsteady diffusion through plant material theory and empirical equation of Ponomaryov had a better fit with the extraction kinetics.

The values of kinetic parameters (k and b) are influenced by various factors, including the specific kinetic models employed, the type of bioactive compound, and the extraction method utilized. However, understanding these relationships can be challenging due to their complexity. Upon comparison, it was observed that the coefficients (k and b) obtained for UAE were consistently higher than those obtained for conventional extraction methods. This underscores the superiority of ultrasonic technology over conventional methods in extracting target bioactive compounds from PFSP. Generally, the washing coefficient (b) tends to have higher values than the slow diffusion coefficient (k). This is because the initial stage of extraction involves rapid transfer of soluble constituents through washing, followed by the slower diffusion stage, during which residual soluble constituents migrate more slowly into the extracting solvent.

## 4. Conclusions

This study successfully designed, optimized, and modeled the extraction of bioactive compounds from PFSP. By employing BBD alongside desirability profiles, the optimal values of time, temperature, and solid-to-liquid ratio (75 min, 70 °C, and 1:15 m/v, respectively, for both response variables) were simultaneously combined to achieve the highest extraction yield of TA and TPC. Utilizing ultrasonic treatment resulted in an 81% increase in the recovery of TA and a 93% increase in the recovery of TPC compared with conventional extraction methods.

Key highlights include the significant reduction in processing time from 24 h in conventional extraction to just 75 min under the optimized conditions of UAE. Moreover, the remarkable stability of the pigments obtained in the process renders them commercially viable as natural colorants for various industries, in addition to their antioxidant properties.

The kinetic study revealed that extraction is influenced by the technology employed and the target bioactive compound. Among the physical models tested, the unsteady diffusion theory and Ponomaryov’s equation models exhibited better fitting with the extraction kinetics. Thus, this study underscores the potential of ultrasound-assisted extraction as a promising, efficient, and rapid technology with low cost, serving as an attractive alternative for extraction of natural colorants from PFSP.

Nevertheless, it is crucial to emphasize the necessity for future studies aimed at elucidating the factors contributing to the enhanced stability of anthocyanins in PFSP compared with other sources of this pigment. Additionally, it entails the adoption of more robust analytical methods for identifying the anthocyanin and phenolic profiles in this raw material, as well as employing more accurate quantification techniques.

## Figures and Tables

**Table 1:** Box–Behnken Design combinations and responses values for total anthocyanin (TA) and total phenolic content (TPC).

Standard Order	Time, X_1_ (min)	Temperature, X_2_ (°C)	Solid-to-Liquid Ratio, X_3_ (g/mL)	TA (mg/100 g)	TPC (mg GAE/100 g)
1	5 (−1)	30 (−1)	1:10 (0)	6.765 ± 0.233	81.416 ± 0.559
2	75 (+1)	30 (−1)	1:10 (0)	11.767 ± 0.529	107.151 ± 1.688
3	5 (−1)	70 (+1)	1:10 (0)	8.202 ± 0.058	85.710 ± 1.091
4	75 (+1)	70 (+1)	1:10 (0)	14.558 ± 0.209	127.527 ± 1.820
5	5 (−1)	50 (0)	1:5 (−1)	9.917 ± 0.029	53.366 ± 1.640
6	75 (+1)	50 (0)	1:5 (−1)	11.334 ± 0.030	72.995 ± 1.090
7	5 (−1)	50 (0)	1:15 (+1)	6.750 ± 0.177	79.971 ± 1.444
8	75 (+1)	50 (0)	1:15 (+1)	16.424 ± 0.435	130.079 ± 1.354
9	40 (0)	30 (−1)	1:5 (−1)	9.979 ± 0.088	74.622 ± 1.205
10	40 (0)	70 (+1)	1:5 (−1)	11.020 ± 0.147	88.822 ± 1.860
11	40 (0)	30 (−1)	1:15 (+1)	12.399 ± 0.177	124.780 ± 1.876
12	40 (0)	70 (+1)	1:15 (+1)	13.833 ± 0.175	130.661 ± 1.757
13	40 (0)	50 (0)	1:10 (0)	9.583 ± 0.354	93.094 ± 1.651
14	40 (0)	50 (0)	1:10 (0)	9.124 ± 0.059	91.135 ± 0.195
15	40 (0)	50 (0)	1:10 (0)	10.270 ± 0.059	95.236 ± 0.335
16	40 (0)	50 (0)	1:10 (0)	9.589 ± 0.468	91.894 ± 1.178
17	40 (0)	50 (0)	1:10 (0)	9.842 ± 0.058	95.074 ± 1.603

**Table 2:** Kinect models of bioactive substances extracted from plant material by solvent.

Model	Kinetic Equation	Linear Transformation
Film theory [18]	$\left(1-\frac{c}{c_{seq}}\right)=\left(1-b\right)e^{-kt}$	$ln\left(1-\frac{c}{c_{seq}}\right)=\ln\left(1-b\right)-kt$
Unsteady diffusion through plant material [18]	$\frac{q}{q_{0}}=\left(1-b\prime \right)e^{-k\prime t}$	$ln\left(\frac{q}{q_{0}}\right)=ln\left(1-b\prime \right)-k\prime t$
Empirical equation of Ponomaryov [18]	$1-\frac{q}{q_{0}}=b\prime \prime +k\prime \prime t$	

Notation: *b*, *b*′, and *b*″—washing coefficient according to the film theory model, the unsteady diffusion model, and the empirical model of Ponomaryov, respectively, 1; *c*—concentration of TA or TPC in liquid extract during the extraction, mg/L; *c_seq_*—maximum concentration of TA or TPC in saturated liquid extract at equilibrium, mg/L; *k* and *k*′—slow extraction coefficient according to the film model and the unsteady diffusion model, min^−1^; *k*″—specific rate of slow extraction according to the empirical model of Ponomaryov, i.e., slope of the dependence of (1 − *q*/*q*_0_) versus time, min^−1^; *q*—content of TA or TPC in plant material during the extraction, mg/100 g or mg GAE/100 g, respectively; *q*_0_—initial content of TA or TPC present in plant material, mg/100 g and GAE/100 g, respectively; and *t*—time, min.

**Table 3:** BBD regression analysis for TA and TPC of purple-fleshed sweet potatoes.

	TA (mg/100 g)		TPC (mg GAE/100 g)	
Variable	Coefficient	*p*-Value	Coefficient	*p*-Value
Constant	11.079	<0.0001 *	96.425	<0.0001 *
*X*_1_	5.613	<0.0001 *	34.323	<0.0001 *
*X*_2_	1.961	0.008 *	9.608	0.046 *
*X*_3_	0.809	0.159	39.118	<0.0001 *
*X*_1_^2^	0.030	0.923	6.727	0.026 *
*X*_2_^2^	−0.671	0.067	−13.891	0.001 *
*X*_3_^2^	−1.455	0.003 *	2.457	0.325
*X*_1_ *X*_2_	0.677	0.313	8.041	0.137
*X*_1_ *X*_3_	4.129	0.0005 *	15.239	0.017 *

BBD—Box–Behnken Design; TA—total anthocyanin; TPC—total phenolic content; *X*_1_—time (min); *X*_2_—temperature (°C); *X*_3_—volume of solid-to-liquid ratio (mL); * *p* < 0.05.

**Table 4:** Values of kinetic parameters.

Model	Process	Total Anthocyanin—TA			Total Phenolic Content—TPC		
		R^2^	k × 10^−3^ (min^−1^)	b (1)	R^2^	k × 10^−3^ (min^−1^)	b (1)
Film theory	UAE	0.899	1.126	0.297	0.884	1.261	0.427
	Conventional	0.592	0.225	0.177	0.501	0.397	0.325
Unsteady diffusion	UAE	0.962	3.051	0.366	0.976	2.187	0.459
	Conventional	0.889	0.349	0.135	0.811	0.487	0.231
Ponomaryov’s equation	UAE	0.937	1.304	0.392	0.961	0.902	0.469
	Conventional	0.882	0.291	0.135	0.799	0.355	0.231

UAE—Ultrasound-assisted extraction; R^2^—coefficient of determination; k—washing coefficient; b—slow diffusion coefficient.

## Data Availability

The original contributions presented in the study are included in the article and Supplementary Material, further inquiries can be directed to the corresponding author.

## Supplementary Materials

The following supporting information can be downloaded at: https://www.mdpi.com/article/10.3390/foods13101497/s1, Table S1. ANOVA for response surface polynomial model off all independent variables; Figure S1. Response surface and contour plots for interactions between time, temperature, and volume of solid:liquid ratio m/v on extraction yield of total anthocyanins (TA). (a) and (b): time and temperature; (c) and (d) time and volume; and (e) and (f) temperature and volume. Figure S2. Response surface and contour plots for interactions between time, temperature, and volume of solid:liquid ratio m/v on extraction yield of total phenolic content (TPC). (a) and (b): time and temperature; (c) and (d) time and volume; and (e) and (f) temperature and volume.
