# Supplementary material for: Design, Optimization, and Modeling Study of Ultrasound-Assisted Extraction of Bioactive Compounds from Purple-Fleshed Sweet Potatoes
Source: Foods. 2024 May 12;13(10):1497. doi: 10.3390/foods13101497 (PMC11119358; doi:10.3390/foods13101497)
Supplement: Supplementary file 1 [file foods-13-01497-s001.zip › foods-2926083-supplementary.pdf]

Article

Design, Optimization, and Modeling Study of Ultrasound-Assisted Extraction of Bioactive Compounds from Purple-Fleshed Sweet Potatoes

Supplementary Material

Table S1. ANOVA for response surface polynomial model off all independent variables.

	TA					TPC				
Variable or Factor	SS	DF	MS	F-value	p-value	SS	DF	MS	F-value	p-value
X_1	63.002	1	63.002	166.332	<0.001*	2356.077	1	2356.077	107.324	<0.001*
X_2	0.004	1	0.004	0.010	0.923	190.554	1	190.554	8.680	0.026*
X_3	5.768	1	5.768	15.229	0.008*	138.476	1	138.476	6.308	0.046*
X_1^2	1.898	1	1.898	5.011	0.066*	812.502	1	812.502	37.011	0.001*
X_2^2	0.980	1	0.980	2.589	0.159	2295.267	1	2295.267	104.554	<0.001*
X_3^2	8.907	1	8.907	23.517	0.003*	25.409	1	25.409	1.157	0.323
X_1X_2	0.459	1	0.459	1.211	0.313	64.658	1	64.658	2.945	0.137
X_1X_3	17.046	1	17.046	45.002	0.001*	232.241	1	232.241	10.579	0.017*
Lack of fit	0.079	2	0.039	0.104	0.903	22.904	2	11.452	0.522	0.618
Pure error	2.273	6	0.379			131.718	6	21.953		
R^2	0.978					0.981				
R^2-adj	0.956					0.960				

X1 – time (min); X2 – temperature (°C); X3 – volume of solid:liquid ratio (mL); TA- total anthocyanin, TPC- total phenolic content; SS, DF and MS represent sum of squares, degree of freedom, and mean square, respectively; *p < 0.05;

Response surface and contour plots for interactions between independent variables (extraction factors) on extraction yield of total anthocyanin from purple-fleshed sweet potatoes:

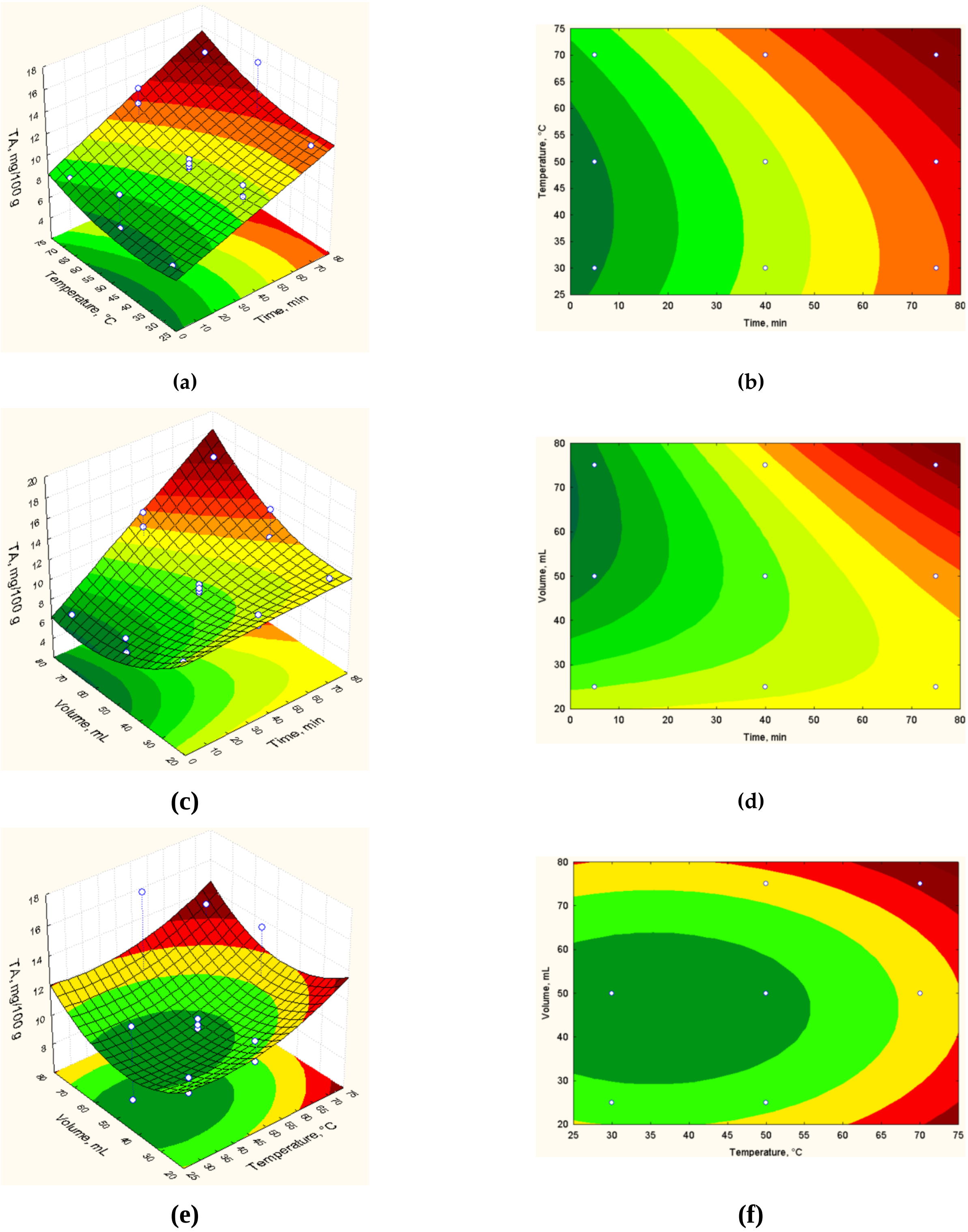

Figure S1. Response surface and contour plots for interactions between time, temperature, and volume of solid:liquid ratio m/v on extraction yield of total anthocyanins (TA). (a) and (b): time and temperature; (c) and (d) time and volume; and (e) and (f) temperature and volume.

Response surface and contour plots for interactions between independent variables (extraction factors) on extraction yield of total phenolic content from purple-fleshed sweet potatoes:

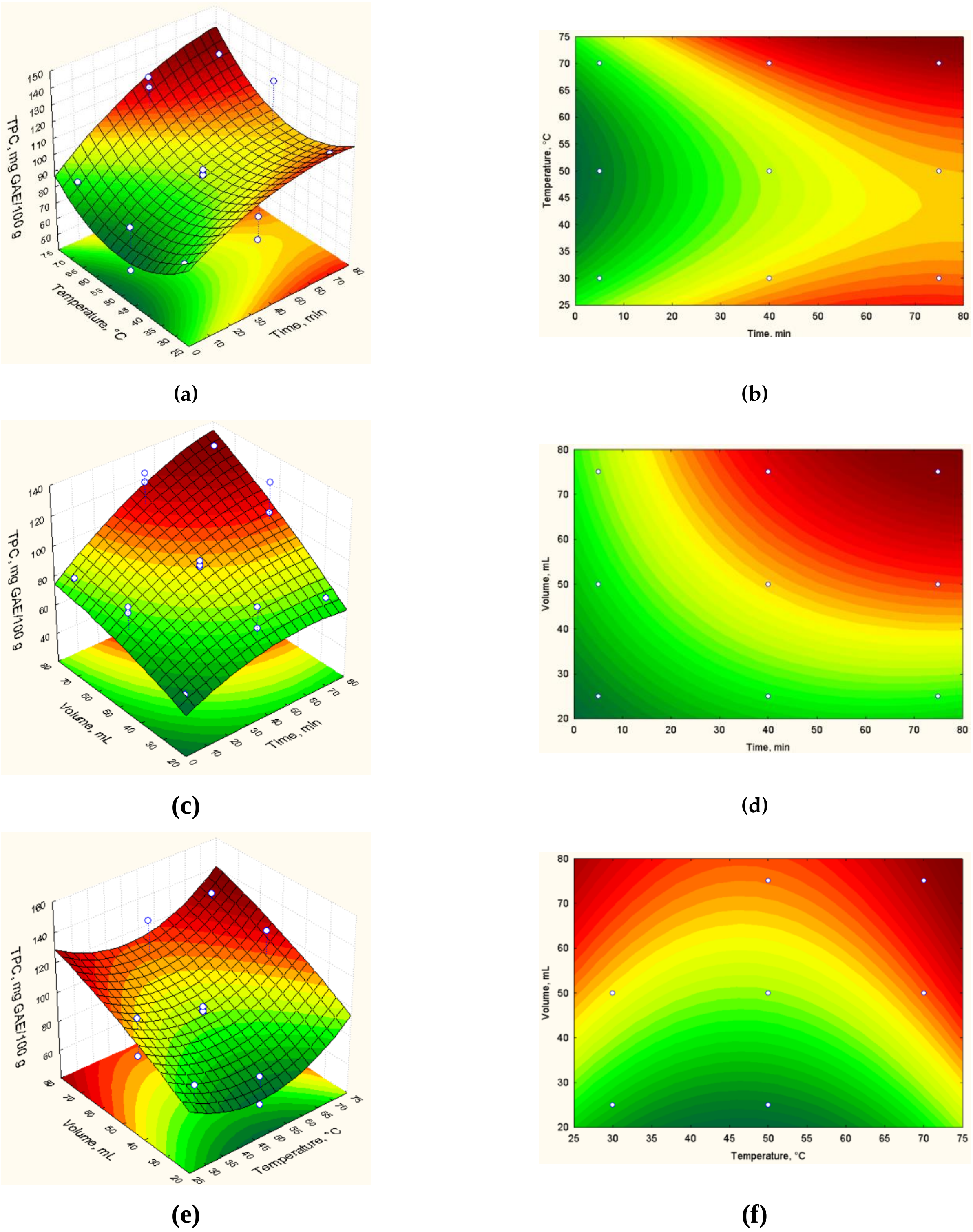

Figure S2. Response surface and contour plots for interactions between time, temperature, and volume of solid:liquid ratio m/v on extraction yield of total phenolic content (TPC). (a) and (b): time and temperature; (c) and (d) time and volume; and (e) and (f) temperature and volume
